# P-917. Management of pediatric focal intracranial infections: opportunities to reduce low-value care

**DOI:** 10.1093/ofid/ofae631.1108

**Published:** 2025-01-29

**Authors:** Lydia S Lu, Allyson Dalby, Preeti Jaggi

**Affiliations:** Children's Healthcare of Atlanta, Atlanta, Georgia; Children’s Healthcare of Atlanta, Atlanta, Georgia; Emory University, Atlanta, GA

## Abstract

**Background:**

Focal intracranial infections are rare but life-threatening complications of sinusitis, otitis media and mastoiditis in children. Management requires collaboration between multiple specialists and prolonged antimicrobial treatment.

**Methods:**

We retrospectively identified patients hospitalized at our tertiary care children’s hospital with focal intracranial infections from Jan 2022 - April 2024 followed in infectious disease clinic. Patient charts were reviewed to inventory imaging, surgical interventions and antibiotic treatment received. We further identified complications in each case requiring intervention, including surgery, hospitalizations and emergency room visits. MRI imaging was defined as low-value if ordered over 2 weeks prior to the study in a patient without new symptoms, or ordered for a hospitalized patient who was afebrile without neurologic symptoms or parental concerns after 30 days of antibiotic therapy.

**Results:**

Thirty-four patients were followed in pediatric infectious disease clinic for outpatient management of: subdural empyema (13, 38%), epidural empyema (11, 33%) and brain abscess (10, 29%). Patients were most likely male (68%) and Black or African American (74%), with a median age of 12 (range: 2-18). Nearly all (94%) had at least one MRI; 19 (56%) had a repeat MRI during hospitalization, 22 (65%) had a repeat MRI after discharge. About one-third of MRIs (31%) after discharge required sedation. In our cohort 18 patients had a low-value MRI (53%) and 5 of these (28%) had more than one low-value MRI. No low-value MRI led to a finding requiring surgical intervention. The mean duration of antibiotic treatment was 42 days (SD 10.8 days). Four patients (12%) returned to the ED while on antibiotic therapy for PICC line issues, with one patient returning 3 times for PICC concerns.

**Conclusion:**

Some management strategies for pediatric focal intracranial infections may be unnecessary or have unrecognized consequences. Repeat MRI for half our cohort was of low-value, exposed many to risks of sedation, and consumed significant family and system resources. Children on prolonged IV antibiotic therapy were at risk for line complications. Routine management of these infections merits reconsideration to decrease unwarranted costs and risks to patient.

**Disclosures:**

**All Authors**: No reported disclosures

